# Old Young People and Young Old People

**Published:** 1881-01

**Authors:** 


### OLD YOUNG PEOPLE, AND YOUNG OLD PEOPLE.

WE meet persons every day whose ages cannot be judged from appearances within five, ten, even twenty years. Two brothers, perhaps, have lived, one forty years and the other forty-two, and yet a stranger might think there were twenty years difference in their ages; the one seeming to be thirty, the other fifty; and in reality there will be that difference in vital power and length of life. We recently saw a notice of the death of a woman in New York, who had lived but forty years, and yet she died from a breaking down of the system, such as occurs in extreme age. It was observed as a remarkable case, and yet the old young and the young old are almost as numerous as the individuals that compose the human race. The causes of this difference are tolerably well understood by most intelligent people; but few heed them; but few are awed by the prospect of retribution, that is as inevitable as death, for every breach of trust in the care of these "earthly temples,' the most wonderful objects of creative power on earth. We are enjoined to preserve our bodies pure and undefiled. There is a sympathy between soul and body that we can never fully comprehend until we have passed to "the other side;" but this we do know, that the mortal can tarnish and soil the immortal. "No man is perfect." But there are degrees of evil ranging from slight faults to unfathomable depths of infamy. No perfectly well man is ever a bad man. Millions of frail and feeble men, hovering between life and death, are of "the salt of the earth." But every bad and vicious man is an invalid with a bias for evil. No man becomes evil in a day.

He commences by little irregularities, that grow into vices, often through thoughtlessness. It is the old, old story. All are weary of hearing it, weary of repeating it; but like the wandering Jew we must march on and do battle against those influences which, if left to themselves, would be destructive to moral and physical well being.

Mr. Emerson says that no man can use tobacco for a month without permanently impairing his health.

The thought is rather startling, and yet it is true. The free use of tobacco for so short a time would affect injuriously the nervous system. No part of the system, once diseased, can ever again be restored to a perfectly healthy conditior. The use of tobacco produces a craving for liquor, so that there are comparatively few persons who use one of these stimulants that do not use both. The use of either deranges the system and makes men prematurely old. When one meets a person who looks ten years older than he did last year—unless he has had severe illness or some special trouble—it is quite safe to suspect that he is drinking, and that he is actually ten years nearer his grave than he was the year before; just as his change of appearance indicates. Life is short enough when it extends to three score and ten, with health and the possession of all the faculties. To voluntarily abridge it by habits and vices that are disgraceful, to be old, infirm and diseased at thirty or forty, indicates a want of self control and of manliness that a sane person should be ashamed of. But the most advanced theory is, that drunkenness is insanity; and with this "mantle of charity" let us cover the faults of those who are beyond hope.

There is one encouraging fact in connection with this subject; and that is, that there is far less drinking among the better and more intelligent young men than formerly. It is to be hoped that the lines will be drawn as closely here as in France, where liquor-drinking is regarded as disgraceful, and but few indulge in it except the most degraded.

### ALLIGATORS' NESTS.

These nests resemble haycocks. They are four feet high, and five in diameter at their bases, being constructed of grass and herbage. First, they deposit one layer of eggs on a floor of mortar, and, having covered this with a stratum of mud herbage eight inches thick, lay another set of eggs upon that, and so on to the top, there being commonly from 160 to 200 eggs in a nest. With their tails they then beat down round the dense grass and reeds, five feet high, to prevent the approach of unseen enemies. The female watches her eggs until they are hatched by the heat of the sun, and then takes her brood under her own care, defending them and providing for their subsistence. Dr. Lutzember, of New Orleans, told the writer that he once packed up one of these nests with the eggs in a box for the museum of St. Petersburg, but he was recommended before he closed it to see that there was no danger of the eggs being hatched on the voyage. On opening one, a young alligator walked out, and he was soon followed by the rest, about 100, which he fed in his house, where they went up and down stairs, whining and barking like young puppies.

